# Viral determinants that drive Enterovirus-A71 fitness and virulence

**DOI:** 10.1080/22221751.2021.1906754

**Published:** 2021-04-09

**Authors:** Pei Yi Ang, Connie Wan Hui Chong, Sylvie Alonso

**Affiliations:** a Infectious Diseases Translational Research Programme, Department of Microbiology&Immunology, Yong Loo Lin School of Medicine, National University of Singapore, Singapore, Singapore; b Immunology programme, Life Sciences Institute, Centre for Life Sciences, National University of Singapore, 28 Medical Drive, Singapore 117456, Singapore

**Keywords:** Hand, Foot and Mouth Disease (HFMD), Coxsackie virus A16 (CVA16), neurovirulence, intertypic recombination, viral quasispecies, RNA-dependent RNA polymerase (RdRp), VP1 capsid, 5’UTR

## Abstract

Hand, Foot and Mouth Disease (HFMD) is usually a self-limiting, mild childhood disease that is caused mainly by Coxsackie virus A16 (CVA16) and Enterovirus A71 (EV-A71), both members of the *Picornaviridae* family. However, recurring HFMD outbreaks and epidemics due to EV-A71 infection in the Western Pacific region, and the propensity of EV-A71 strains to cause severe neurological complications have made this neurotropic virus a serious public health concern in afflicted countries. High mutation rate leading to viral quasispecies combined with frequent intra- and inter-typic recombination events amongst co-circulating EV-A71 strains have contributed to the great diversity and fast evolution of EV-A71 genomes, making impossible any accurate prediction of the next epidemic strain. Comparative genome sequence analyses and mutagenesis approaches have led to the identification of a number of viral determinants involved in EV-A71 fitness and virulence. These viral determinants include amino acid residues located in the structural proteins of the virus, affecting attachment to the host cell surface, receptor binding, and uncoating events. Critical residues in non-structural proteins have also been identified, including 2C, 3A, 3C proteases and the RNA-dependent RNA polymerase. Finally, mutations altering key secondary structures in the 5’ untranslated region were also found to influence EV-A71 fitness and virulence. While our current understanding of EV-A71 pathogenesis remains fragmented, these studies may help in the rational design of effective treatments and broadly protective vaccine candidates.

## 1. Introduction to HFMD and Enterovirus-A71

Enterovirus-A71 (EV-A71) is the second most common causative agent after Coxsackievirus A16 (CVA16) of Hand, Foot and Mouth Disease (HFMD), a childhood disease that occurs worldwide but prominently affects countries within the Asia-Pacific region including China, Taiwan, Vietnam, Thailand, Malaysia, Singapore, Japan, Korea and Australia [1]. Numerous outbreaks and cyclical epidemics have been reported in these countries, with the latest EV-A71 outbreak in Singapore in 2018 during which 1,249 cases were reported within a single week [2]. Infections are usually followed by fever and sore throat before occurrence of characteristic blisters and lesions on the palms, soles and oral mucosa [3]. HFMD is highly transmissible via bodily fluids or contaminated objects and even asymptomatic adults are able to transmit the virus [3].

While HFMD is usually a self-limiting disease, serious complications have been reported with involvement of the central nervous system (CNS), including aseptic meningitis, brainstem encephalitis, acute flaccid paralysis and cardiopulmonary dysfunction of neurogenic origin [4]. When not fatal, HFMD-associated neurological complications may lead to long-term cognitive and motor disorders [5]. Up to 90% of HFMD cases with neurological complications have been attributed to EV-A71 infection [4]. This makes EV-A71 an important neurotropic virus after the eradication of its close cousin poliovirus (PV) from most of the surface of our planet. Three vaccines have made it to the Chinese market so far [1]. They are inactivated whole virus vaccines that cover a single genogroup (C4). These monovalent vaccines however, may not confer long-term pan-genogroup protection, and may influence epidemiological dynamics of EV-A71 strains [6]. Hence, efforts to develop multivalent vaccines including various EV-A71 genogroups and CVA16 have been pursued [7].

**Figure 1.**
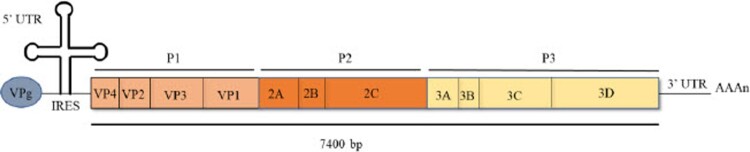
Schematic illustration of EV-A71 genome organization.

A member of the *Picornaviridae* family, EV-A71 is a non-enveloped, positive sense, single-stranded RNA virus. Its genome is approximately 7.4kb-long and the coding region, which encompasses sub-regions P1, P2 and P3 is preceded by a short, structured 5’ untranslated region (UTR), while the 3’UTR ends with a ploy-A tail (Figure 1). The P1 region codes for four structural proteins VP1 to VP4, which make up the protein capsid where the exposed VP1-3 are antigenic. The P2 and P3 regions code for seven non-structural proteins that include viral proteases and the RNA-dependent RNA polymerase (RdRp). The infection cycle of EV-A71 in its host cell starts with receptor-mediated entry involving the capsid protein VP1, followed by uncoating that allows release of the viral genome into the cytoplasm. After a first round of translation to produce structural and non-structural proteins, the viral genome is replicated followed by virus assembly and maturation, before the newly formed virions exit the cells upon apoptosis-induced cell lysis or via non-lytic exit pathway [8]. The role of each viral proteins during EV-A71 infection cycle has been recently reviewed elsewhere [9].

Infection outcome typically results from the interplay between the intrinsic virulence of the pathogen, and the genetic make-up and immune status of the host. This review focuses on the viral determinants that play a critical role in EV-A71 fitness and virulence. An earlier study on the circulation of EV-A71 lineages in different countries and geographical areas since 1960 indicated that no association could be drawn between a particular genogroup or subgenogroup and the increased risk of neurological complications [10]. More recently however, complete genome analyses of EV-A71 strains have been conducted to identify genetic determinants responsible for different clinical patterns. Nucleotide differences in the 5’-UTR of the viral genome, and amino acid changes in viral proteins have been proposed to play a role in virulence. Additionally, mutagenesis studies have allowed explore the role of a number of nucleotides or amino acids in EV-A71 fitness and virulence, both *in vitro* and in animal models. We provide here an overview of these latest findings and the gaps in our knowledge that remain to be addressed.

## 2. Mechanisms driving EV-A71 strains diversity

### 2.1. EV-A71 strains classification and circulation

EV-A71 strains have been isolated in various countries all over the world, and have been classified into genogroups (A-G) and subgenogroups, based on their VP1 gene sequence similarity. A Bayesian tree was generated that traced the time of origin and evolutionary history of these strain**s** [11]. Furthermore, reports of EV-A71 outbreaks worldwide have allowed monitor the circulation and evolution of EV-A71 strains over time [12]. Extensive and rapid shifts in genogroup and subgenogroup dominance have been noted. Intra-genogroup dominance shifts have also been reported over time in a single country. Furthermore, it is important to note that within a single outbreak, co-circulation of several genogroups and subgenogroups is common [12]. China, however, has seen persistent predominance of subgenogroup C4 over the past 15 years, and represents more the exception than the rule. The introduction of three monovalent C4 vaccines in China may however drive strain replacement and lead to a shift in EV-A71 genogroup or subgenogroup dominance, as seen with other infectious diseases [13].

Observation of rapid shifts in EVA-71 genogroup or subgenogroup dominance in the same country or geographical location is likely facilitated by the fact that several lineages co-circulate at any one point of time and take turn to become predominant. Herd immunity may represent an important factor that drives the shift in genogroup prevalence. Additionally, evidence of sequence recombination between EV-A71 strains, as well as spontaneous mutations in the viral genome contribute to the genetic diversity and drive evolution of EV-A71 strains.

### 2.2. Recombination

Studies employing recombination analysis techniques found evidence that strongly supported horizontal acquirement of sequences for almost every tested EV-A71 strains [14,15]. Sequence analysis of a single subgenogroup revealed that a group of isolates with similar sequences sampled from a single location were interspersed with sequences from strains found in geographically distant areas [15].

Recombination events in EV-A71 can be intra- or intertypic and occur non-randomly in both structural and non-structural protein gene sequences, or at the 5’ UTR, and with the highest frequency found in the 3D region that encodes for the RdRp [14,16]. Recombination events appear to drive the fitness and virulence of EV-A71 strains, and lead to the emergence of new EVA-71 strains responsible for major outbreaks. An EV-A71 C4 strain that had undergone recombination with CVA16 in the 3D region was responsible for fatal outbreaks in China where neurological complications were reported [17]. A similar observation was made upon the analysis of HFMD cases in China between 2011 and 2012, where viruses resulting from recombination between EV-A71 and CVA16 were isolated in severe cases [18]. In addition, a double recombination event between EV-A71 genogroups B and C, and a CVA16 strain was suspected to be the cause of an epidemic in China [19]. Intertypic recombination events in EV-A71 are not limited to its close relative CVA16, as recombination with other Human Enterovirus-A (HEA) strains has also been reported [20]. Copy-choice recombination, also known as template switching, can also occur when RdRp switches templates during synthesis, resulting in a mosaic-like genome [21]. This has been reported in PV, and could potentially occur in EV-A71 as well [22].

### 2.3. Spontaneous mutations and quasispecies

The P2 and P3 regions coding for non-structural proteins in enteroviruses are expected to be sites where recombination occurs most frequently due to their higher degree of sequence homology. In contrast, the more variable P1 region, coding for the viral capsid proteins is believed to undergo mutations more frequently, particularly at the VP1 region as interactions with antibodies and host cell receptors require the virus to evolve quickly in order to evade the host immune system while maintaining its ability to bind to its host receptors [19]. Mutations occur at high rates in RNA viruses due to the lack of proofreading activity in the RdRp, which leads to approximately 1 × 10^−4^ substitutions per nucleotide copied [23]. Such high mutation rate allows EV-A71 to adapt rapidly to selection pressures, which select for beneficial mutations [24]. A study on the potential antiviral activity of two capsid-binding compounds showed that EV-A71 acquired resistance mutations in VP1 upon successive passages in the presence of the compounds [25]. The resistance mutations acquired however, had a fitness cost and they were quickly lost upon removal of the selection pressure, highlighting the high plasticity of EV-A71 genome. The ability of EV-A71 to rapidly acquire mutations in response to specific culture conditions has also been documented in another study where EV-A71 acquired four mutations in VP1 sequence at position 104, 145, 146, and 241 that facilitated its replication after only three sub-passages in Rhabdomyosarcoma (RD) cells [26]. Mutations in the P1 region can also improve viral fitness by promoting interaction with attachment receptors such as heparan sulfate (HS) or conferring resistance to capsid-targeting drugs [27–29]. Mutations driven by selection pressure are not strictly limited to the P1 region. A point mutation in the RdRp from EV-A71 was reported to confer resistance to Ribavirin, a broad range antiviral compound effective against a variety of RNA viruses [30]. Ribavirin is a nucleoside analogue that is mistakenly incorporated into the nascent RNA strand and stops RNA synthesis [35]. The point mutation in RdRp was found to increase fidelity of the enzyme, hence avoiding incorporation of the nucleoside analogue [31].

In addition, the error-prone RdRp of enteroviruses resulting in high mutation rates enables the formation of quasispecies, where the viral progeny consists of a spectrum of closely related genome variants [32]. The existence of quasispecies in enteroviruses has been well documented and is evidenced by the formation of plaques of different sizes from a single viral culture suspension [33]. Importantly, the genetic diversity arising from quasispecies dynamics was found to play a critical role in the virus fitness and virulence [24,31]. Consistently, higher-fidelity RdRp EV-A71 and PV mutants, which limited the generation of quasispecies, were found to be attenuated *in vivo* [31,34]. The genetic diversity resulting from quasispecies dynamics was recently proposed to have driven the development of neurovirulent EV-A71 isolates in humans [35]. Similarly, genetic diversity in PV was demonstrated to be critical for neuroinvasion in order to overcome the host antiviral activities triggered upon entry of the first few viral particles into the CNS [34]. This was further supported by findings that high fidelity PV mutants were less neurovirulent and pathogenic, or elicited higher titres of neutralizing antibodies [36].

## 3. Molecular determinants that influence EVA-71 fitness and virulence

The dynamic changes occurring in the genome of EVA-71 resulting from recombination events and mutations have led to the emergence of strains with distinct fitness and virulence, and a distinct ability to cause neurological disease in their host. Earlier studies have reported that EV-A71 isolates that caused neurological complications displayed greater resistance to high temperature and enhanced replication capacity compared to milder strains [37–39]. These features were proposed to help the virus survive in its host during the febrile stage and ensure successful neuroinvasion and replication in the CNS. Temperature-adapted strains were obtained in *in vitro* culture systems, supporting that the temperature-resistant phenotype could be acquired through mutation [40].

More recently, comparative genome sequence analyses and mutagenesis approaches have allowed the identification of mutations in both structural and non-structural proteins, as well as in the UTRs that significantly influenced EV-A71 fitness and virulence (Table 1). In our literature survey, we have employed the following keywords: Enterovirus 71 virulence; molecular determinant; EV-A71 mutation affecting virulence; comparative studies on EV-A71.

**Table 1.** Mutations in EV-A71 genome that influence fitness and virulence.

		Phenotype/Mechanism			
Region	Mutation	*In vitro*		*In vivo*	References
VP1	31G	**DLD-1, RD and SK-N-SH cells** ➢Reduced virion stability at 35°C, 36°C, 37°C, and 39°C ➢Reduced viral titer in DLD-1 cells ➢Increased growth in SK-N-SH cells	–		[35]
	L97R	**SH-SY5Y cells** ➢Replicative advantage ➢Improved binding to host cells ➢Associated with E167G/A (Entry, or assembly)	–		[66]
	E98K	**L929 and Neuro-2a cells** ➢Improved growth kinetic	**Two-week-old (BALB/c mice)** ➢Non-fatal ➢No visible damage to liver and lungs and were comparable to control mice infected with PBS		[67]
	107A	**Vero cells** ➢Reduced viral growth and titre ➢Regulates the cleavage of precursor VP0 and potentially affects virus uncoating and maturation	–		[50]
	145G	**RD cells overexpressing hSCARB2** ➢Improved replication ➢Enhanced HS binding compared to 145E	**Neonatal mice (ICR mice)** ➢Lower viral loads in organs ➢Non-fatal **hSCARB2 transgenic mice** ➢High adsorption to non-susceptible cells and rapid decrease in plasma of hSCARB2 transgenic mice ➢Lowered viral load in organs ➢Non-fatal		[44]
	145E	**RD cells overexpressing hSCARB2** ➢Lower virus titer produced in mutants compared to 145G	**Neonatal mice (ICR)** ➢Higher viral load in organs ➢Lethal in mice **hSCARB2 transgenic mice** ➢More efficient replication and greater neuroinvasion compared to 145G		
		**RD cells** ➢Mouse-adapted strain ➢Lower titers compared to parental strain ➢Decreased binding to cell	**1-week old mice (BALB/c)** ➢Caused 100% mortality		[45,46]
		**Jurkat T cells** ➢Molecular switch that changes virus from PSGL-binding (145G/Q) to non-PSGL-binding	–		[68]
		–	**5-day-old BALB/c mice** Caused 100% mortality		[69]
		–	**Cynomolgous monkeys** ➢CNS inflammation and damage ➢Neurological manifestations (tremors) ➢145E mutants recovered from monkeys inoculated with 145G mutant, while those inoculated with 145E maintained the same mutation, suggesting a selection pressure for 145E. ➢Innate immune system response with rise in IFNα, TNFα and IL-6 ➢More resistant to neutralizing antibodies		[47]
	Double mutant VP1 145E & VP2 149M	**Neuro-2a cells** ➢Higher viral titers ➢Higher viral protein expression	**1-day old mice (ICR)** ➢149M increased effect of 145E for mouse lethality		[48]
	Double mutant 145E & 98E	**Murine NIH/3T3 and Neuro-2a cells** ➢Productive infection ➢Addition of 169F mutation further enhanced infectivity	**1-week old mice (BALB/c)** ➢No significant signs of disease and non-lethal in mice		[51]
	K162A	**Computer simulated Heparin** ➢Reduced strength of interaction with heparin	–		[28]
	192M	**Vero cells** ➢Resistance to EV-A71 inhibitor pyridyl imidazolidinone	–		[27]
	K215A	**Vero cells** Increased thermostability where infectivity of mutant is higher than wild type after treatment at 42^0^C	–		[70]
	K242A	**Computer simulated Heparin** ➢Reduced strength of interaction with heparin **RD cells** ➢Reduced binding on cell surface	–		[28]
	K244A				
	K244E	–	**1-day-old (BALB/c mice)** ➢Increased virulence **5-day-old (BALB/c mice)** ➢Caused 100% mortality at virus doses greater than 8.7 × 10^1^ TCID_50_ **6-week-old (AG129 mice)** ➢Increased virulence		[71] [72]
	A289T	**Human Brain Microvascular Endothelial Cells (HBMECs)** ➢Decreased viral attachment, replication, protein synthesis, and virus particle secretion.	**1-week-old (BALB/c&Sv129)** ➢Decreased morbidity; decreased CNS infectivity		[73]
	Q172A	**RD cells** ➢Loss in both binding and infection	–		[49]
	Q152A R166A W171A T173A T175A N176A S178A F180A R236A	**RD cells** ➢Loss in binding and infection ➢No impairment of viral assembly except for N176A	–		
VP2	K149I	**Chinese Hamster Ovary cells** ➢More efficient replication with higher virus titre	–		[45]
5’ UTR	C158U	–	**Three-day-old ICR mice** ➢Prolonged survival of infected mice ➢Reduced viral replication and virulence		[53]
	C115T	–	**1-day old BALB/c mice** ➢Reduced virulence		[60]
	A158T C475T A486G G487A	**RD cells** ➢Reduced CPE ➢Lower viral titers	–		[65]
Helicase 2C	K135A D176N C270A C281A C286A I141R S282R E325A L327K F328A F328Y	**Vero cells** ➢Reduced viral production ➢Reduced ATPase activity of protein 2C	–		[74]
Protease 3A	A5262G	**RD cells** ➢Absence of CPE ➢Reduced viral titer	–		[65]
Protease 3C	N69D	**RD cells** ➢Decreased enzymatic/catalytic activity ➢Decreased viral production	–		[75]
	T79V	**RD cells** ➢Increased viral replication **Immunoprecipitation** ➢Increased interaction with TRIM21	–		[58]
	R84Q	**Vero cells** ➢Loss of RNA-binding activity ➢No plaque formation at 8 d.p.i.	–		[76]
	I86A	**Vero cells** ➢Loss of proteolytic activity ➢No plaque formation at 8 d.p.i.	–		[76]
3D polymerase	I251T	**SK-N-SH cells** ➢Viral replication rate reduced by up to 100-fold at 39.5°C	**ICR (newborn)** ➢Reduced virulence		[77]
	L123F	**RD cells** ➢Increased replication fidelity ➢In vitro growth not altered	**Ten-day-old (AG129 mice)** ➢Reduced virulence ➢Reduced viral titers		[31]
	Double mutation G64R & L123F	**RD cells** ➢Increased replication fidelity ➢Attenuated in vitro growth	**Ten-day-old (AG129 mice)** ➢Reduced virulence ➢Reduced viral titer compared to L123F single mutation.		[31]

### 3.1. Mutagenesis approaches

#### 3.1.1. Structural proteins VP1-4

Mutations that influence EV-A71 binding and entry through interaction with cellular receptors have been found to play an important role in EV-A71 infectivity and virulence. EV-A71 entry into human cells is primarily mediated by SCARB2 and PSGL-1 while other surface molecules such as Annexin2 and HS function as attachment receptors [41]. Specifically, HS has garnered a lot of interest with recent studies highlighting the important role of this surface attachment receptor in EV-A71 virulence and pathogenesis [29], and the potential antiviral activity of HS mimetic compounds [42].

The receptor binding domain lies within the VP1 capsid protein, and numerous mutations in this region have been found to affect receptor binding [41], with consequences on cell/tissue tropism and replication efficacy both *in vitro* and/or *in vivo* (Table 1). Mutations in VP1 alter capsid-receptor interactions either directly by changing the charge of the critical residues, or indirectly by changing the orientation of the critical residues when the neighbouring residues are mutated [28]. Other VP1 mutations were found to destabilize the capsid, impair VP1-VP2 interactions and affect virus uncoating [43].

One notable residue is VP1 145, which resides within the DE loop and is surrounded by positively charged residues. A glycine (G) residue at that position increased significantly EV-A71 binding affinity to HS due to the highly positive charge of the five-fold pentamer, which facilitates electrostatic interactions with HS [43,44]. In contrast, presence of the negatively charged glutamate (E) at position 145 in VP1 led to decreased binding affinity to HS, resulting in lower viral titres in the culture supernatant [28, 44–46]. However, VP1 145E mutant surprisingly performed better *in vivo* compared to VP1 145G virus as evidenced by higher viral loads and increased neuroinvasion in neonatal mice and cynomolgous monkeys [44,47]. The seemingly contradictory *in vitro* and *in vivo* observations led the authors to propose that the increased adherence property of VP1 145G virus to HS-expressing cells limited its ability to disseminate and invade the CNS [43,44]. In addition, Huang *et al* reported increased virulence with a VP1 145E / VP2 149M double mutant, supporting cooperative interaction between VP2 and VP1 during the entry step [48].

Other VP1 mutations have also been reported to alter binding efficacy to surface receptors. *In silico* simulation models predicted that replacement of lysine (K) residues with alanine (A) at VP1 162, 242 or 244 would lead to reduced strength of the electrostatic interactions with HS, and this was experimentally confirmed in RD cells [28]. Furthermore, although a number of key residues in VP1 have been found to play a critical role in the viral entry step including attachment and uncoating [49–51], the molecular and structural insights are lacking to explain their role.

It is important to note that EV-A71 displays weak binding affinity to murine SCARB2 (mSCARB2) and PSGL-1 (mPSGL-1), and it is likely that the virus uses different surface entry receptors in mice [48,51]. Therefore, whether the findings on the role of various VP1/VP2 mutations in EV-A71 virulence in mouse models hold true in human settings remains to be demonstrated. Some of these findings however were generated in hSCARB2 transgenic mice [44] or in NHP models [47], the latter share high homology with hSCARB2 and are thus expected to be more predictive.

#### 3.1.2. Non-structural proteins and UTRs

While capsid proteins primarily drive virulence by influencing interactions with the host receptors, mutations within non-structural proteins are expected to affect virulence by altering intracellular steps of the infection cycle such as genome replication, protein translation, and virus assembly and maturation. Mutations in proteins 2C, 3A, 3C, and in 3D polymerase have been reported to influence the *in vitro* fitness of EV-A71 strains (Table 1). Mutations within UTRs of the viral genome could also affect EV-A71 fitness and virulence (Table 1). For example, nucleotide changes that affect the stem loop structure in the 5’UTR were associated with reduced IRES-dependent translational activity [52]. A single point mutation at position 158 (C158U) in domain V of the stem loop structure led to reduced virulence of an EV-A71 strain from genogroup B1 in three-day-old ICR mice [53]. Interestingly, the same C158U substitution was associated with a fatal case, highlighting the lack of direct correlation between virulence in a mouse model and clinical outcome in patients [54]. In PV, C158U substitution was predicted to cause a change in the secondary structure of the upper stem loop II, which altered binding affinity to host factors, and subsequently attenuated viral genome replication [55,56]. Similar results were obtained with B4 genogroup strains where A158T mutants caused minimal CPE and led to reduced viral RNA copy number, plaque forming units (pfu) and VP1 production in RD cells [57].

In addition, polymorphism at the 79th amino acid in EV-A71 3C protease was associated with varying levels of clinical severity [58]. Mechanistically, the study found that T79V substitution in 3C led to higher viral replication while reducing interaction with TRIM21, a component of antibody-dependent intracellular neutralization.

### 3.2. Comparative genome sequences analyses

In addition to experimental mutagenesis approaches, numerous genome comparative studies have been conducted over the years. While these studies failed to correlate a particular genogroup with disease severity [59], they have allowed identify viral determinants of EV-A71 virulence in its human host. Upon comparing clinical isolates that led to mild versus more severe disease outcome in patients, differences were observed in virtually all parts of EV-A71 genome, including coding and non-coding regions. In some cases, follow up experiments have been conducted to verify the potential role of the viral determinants identified (Table 2**)**. For example, a single nucleotide difference located in the 5’ UTR stem loop structure at position 115 between two EV-A71 strains of different clinical severity was associated with a differential virulence phenotype in BALB/c mouse neonates [60]. In another study, Li et al [38] replaced the protease 2A region from a severe EV-A71 strain with that of a milder strain. The recombinant virus displayed slower replication rate compared to the severe strain at two different temperatures (37 and 39.5 °C) and in three different cell lines. One-day-old ICR mice infected with this mutant also showed less severe pathological features and lower viral loads compared to mice infected with the severe strain [38]. Three amino acid differences at positions 64, 68 and 85 were mapped between both protease 2A sequences. However, the study did not investigate further the mechanisms involved in the differential virulence phenotype observed, and the relative contribution of each of these residues.

**Table 2.** Comparative studies of EV-A71 genome sequences and their phenotypic differences*.*

	Phenotypic Differences		
Mutations	*In vitro*	*In vivo*	References
Protease 2A from a severe strain → protease 2A from a mild strain **Protease 2A** Position 64 (Y64H) Position 68 (M68R) Position 85 (D85E)	**RD cells, Vero cells and SH-SY5Y cells** ➢Slower replication compared to the severe strain at 37 °C and 39.5 °C ➢Less viral RNA compared to the severe strain at 37 °C and 39.5 °C	**1-day-old ICR mice** ➢Less severe pathological changes compared to severe strain ➢Lower viral load compared to severe strain	[78]
3CD region from severe strain → 3CD region from mild strain **Protease 3C** Position 138 (V138I) **3D polymerase** Position 37 (S37N) Position 143 (R143K) Position 176 (I176V) Position 261 (E261G) Position 360 (G360A)	**RD cells and Vero cells** ➢Milder CPE as compared to severe strain at 37 °C and 39.5 °C ➢Decreased replication rate compared to severe strain at 39.5 °C	**1-day-old ICR mice** ➢Mice showed no paralysis, no death, less body weight loss, and milder symptoms as compared to the mice injected with the severe strain ➢Lower viral load as compared to mice infected with the severe strain	[38]
Weakly pathogenic strain → Highly pathogenic strain **5’ UTR** Position 115 (115C>T) Position 132 (132T>C) Position 811 (811T>C) **VP1** Position 639 (G639S) **VP3** Position 485 (Q485R) Position 504 (A504V) Position 537 (V537I) **Protease 2A** Position 937 (G937S) **Protease 2B** Position 1014 (A1014V) Position 1045 (I1045V) Position 1062 (T1062I) **Protease 2C** Position 1135 (K1135R) Position 1145 (E1145D) Position 1155 (V1155A) Position 1361 (V1361M) **Protease 3C** Position 1597 (I1597V) Position 1617 (D1617N) **3D Polymerase** Position 1746 (L1746F) Position 2146 (A2146V) **3’ UTR** Position 7347 (7347A>G)	Nucleotide differences when a weakly pathogenic strain (JN200803) is compared to a highly pathogenic strain (JN200804) **MA104 cells** ➢Both strains (weakly and highly pathogenic strain) exhibited similar CPE ➢No difference in virulence as determined by TCID_50_ titration	**1-day-old BALB/c mice** ➢Mice infected with the highly pathogenic strain exhibited hind limb paralysis by day 4 post-infection. ➢Mice infected with the weakly pathogenic strain had no evident neurological symptoms and remained healthy.	[79]

## 4. Concluding remarks and future perspectives

This review provided an update on the main viral determinants that have been found to influence EV-A71 fitness and virulence. Unsurprisingly, majority of the residues identified mapped in the main capsid protein VP1, affecting attachment or receptor binding. A number of mutations in non-structural proteins have also been described to influence *in vitro* replication of the virus but their *in vivo* phenotype has yet to be reported. Nevertheless, this incomplete picture still provides useful information and helps improve our understanding of EV-A71 virulence mechanisms.

Despite its public health significance and economic impact on afflicted societies, HFMD has remained under-studied. Great majority of the research efforts have been led by academic institutions supported by governmental funding, as HFMD is not part of pharmaceutical companies’ R&D portfolio. As a result, patchy knowledge and limited understanding of the virulence mechanisms have hampered the development of effective treatments and the rational design of vaccine candidates. The limited availability of relevant animal models [61] has also contributed to the difficulty in translating pre-clinical findings.

Furthermore, while the ability to invade the CNS represents the cornerstone of EV-A71 neurovirulence, an increasing body of evidence has underscored the importance of the host immune response in EV-A71 pathogenesis. Indeed, severe HFMD cases have been associated with heightened production of pro-inflammatory mediators in EV-A71 patients with CNS complications [62]. EV-A71 immunopathogenesis has not been fully elucidated yet, although a number of viral proteins have been identified to interact with, and manipulate the host immune system [63]. Thus, it appears that the ability of EV-A71 to manipulate its host immune system represents an integral part of its virulence strategy, and influences the clinical outcome. However, while some recent evidence support that EV-A71 strains associated with severe clinical outcome manipulate differently their host immune signalling pathways [64], the viral determinants involved have yet to be reported.

Beyond whole inactivated virus formulations, the identification of key viral determinants involved in EV-A71 neurovirulence and immunopathogenesis represents an opportunity to develop live attenuated vaccine candidates [65] that may offer complete and strong protection upon a single immunization, which would improve the vaccine take up rate and control more effectively HFMD epidemics.
